# *Wolbachia* interferes with Zika virus replication by hijacking cholesterol metabolism in mosquito cells

**DOI:** 10.1128/spectrum.02180-23

**Published:** 2023-10-09

**Authors:** Brent Edwards, Elodie Ghedin, Denis Voronin

**Affiliations:** 1 Systems Genomics Section, Laboratory of Parasitic Diseases, National Institute of Allergy and Infectious Diseases, National Institutes of Health, Bethesda, Maryland, USA; University of Nebraska Medical Center, Omaha, Nebraska, USA

**Keywords:** *Wolbachia*, Zika virus, cholesterol, *Aedes*

## Abstract

Zika virus is a member of the arbovirus *Flaviviridae* family transmitted by *Aedes* mosquitos and it is associated with microcephaly in infants born to infected mothers. *Wolbachia* is an intracellular gram-negative alpha-proteobacteria that infects many species of arthropods, including mosquitos. The presence of *Wolbachia* in mosquitos has been shown to control the vector population and suppress arbovirus transmission. One mechanism of *Wolbachia*-mediated interference with virus replication is competition over host resources between *Wolbachia* and the virus. We hypothesize that cholesterol metabolism is involved in *Wolbachia*-mediated virus suppression due to its important role in Zika virus replication. In this study, we determined that *Wolbachia* impacted virus replication by altering cholesterol biosynthesis in *Aedes albopictus* C6/36 cells, diverting resources from the host cell mevalonate (MVA) pathway to fulfill the needs of the bacteria. This resulted in a decrease of total cholesterol, increased *Wolbachia* loads, and decreased viral titers. Inhibition of the MVA pathway using fluvastatin decreased total cholesterol and viral titers, mimicking the effects of *Wolbachia* on the virus in *Wolbachia-*free cells. We also found that *Wolbachia-*infected cells had depleted lipid droplets, the main component of which is cholesterol esters. We confirmed that cholesterol esterases were upregulated in response to virus infection in C6/36 cells. Functional analysis showed that alteration of cholesterol metabolism simulated *Wolbachia*-mediated inhibition of virus infection in C6/36 cells. Our study provides a mechanism behind *Wolbachia*-induced interference of arbovirus replication and could help advance strategies to control arbovirus pathogens in insect vectors and human infections.

**IMPORTANCE:**

Arthropod-borne viruses are emerging pathogens that are spread widely by mosquitos. Zika virus is an arbovirus that can infect humans and be transmitted from an infected mother to the fetus, potentially leading to microcephaly in infants. One promising strategy to prevent disease caused by arboviruses is to target the insect vector population. Recent field studies have shown that mosquito populations infected with *Wolbachia* bacteria suppress arbovirus replication and transmission. Here, we describe how intracellular bacteria redirect resources within their host cells and suppress Zika virus replication at the cellular level. Understanding the mechanism behind *Wolbachia*-induced interference of arbovirus replication could help advance strategies to control arbovirus pathogens in insect vectors and human populations.

## INTRODUCTION

*Wolbachia* is an intracellular gram-negative alpha-proteobacteria that infects many species of arthropods and filarial nematodes of veterinary and medical importance. The release of *Wolbachia*-infected mosquitos has been used as a natural tool to change insect populations and control arbovirus transmission by mosquitos. Cytoplasmic incompatibility (CI) is the phenomenon by which *Wolbachia* infection can directly control mosquito populations when male *Wolbachia-*infected mosquitos mate with non-*Wolbachia*-infected females, producing non-viable embryos (1). Vector control efforts have exploited CI by releasing *Wolbachia-*infected male mosquitos into the wild, resulting in vector population decline (2). *Wolbachia* infection also diminishes virus transmission from vectors by affecting mosquito physiological processes (3, 4). On the organismal level, mosquito infection with *w*MelPop, a strain of *Wolbachia* that replicates at a high rate, decreases mosquito blood meal volume (5) and shortens the mosquito lifespan, resulting in lower viral load in the mosquito (6). Infection titers for several arboviruses [including Zika virus (ZikV), dengue virus (DENV), and West Nile virus] were found to be decreased in several mosquito cell lines infected with *Wolbachia* as compared to *Wolbachia-*free mosquito cells (7 – 9). These findings support the strategy of mosquito control, where both male and female *w*MelPop*-*infected mosquitos are released into the environment (1). Due to CI, the percentage of mosquitos infected with *w*MelPop is rising, thus limiting arbovirus replication within the mosquitos and interfering with disease transmission (1).

Many studies have concluded that *Wolbachia* and arbovirus competition for host resources is responsible for reduced viral loads in *Wolbachia-*infected cells and insects (4, 10). Here, we hypothesize that competition over resources related to cholesterol metabolism may be involved in *Wolbachia*-mediated suppression due to the important role of cholesterol and its precursors in intracellular bacteria and ZikV replication. For obligate intracellular bacteria, cholesterol is used as the primary component of bacterial membranes. For the host cell, the presence of bacteria perturbs intracellular cholesterol trafficking and lipid metabolism (11 – 13). It has been shown in humans that intracellular pathogenic bacteria increase the ability of infected host cells to accumulate cholesterol, thus evading the host immune response (14, 15). Production of cholesterol, its precursors, and secondary metabolites are crucial for intracellular bacteria, as chemical intervention of these processes suppresses bacterial infection of mammalian cells (16).

Cholesterol metabolism is essential for arbovirus infection and replication in both arthropod and mammalian hosts (17, 18). ZikV modulates host cell lipid metabolism by using membrane components of the endoplasmic reticulum (ER) to form the replication complex (19, 20). This replication complex generates new viral particles and helps with immune evasion (21). Additionally, infection with arboviruses, such as DENV, increases the activity of fatty acid synthase, which increases cholesterol synthesis within the replication complex (17, 22, 23). Besides providing the substrate for the bacterial replication complex, stored cholesterol in lipid droplets (LDs) plays an important role in viral replication (24, 25). LDs are the primary energy storage method for the cell where cholesterol is stored in its esterified form (26). In addition, during arbovirus replication, the capsid protein of the virus accumulates on LDs and is then mobilized to the viral assembly site near the ER (25, 27, 28). Cholesterol uptake increases upon DENV infection through the increased expression of the low-density lipoprotein receptor and scavenger receptor class B type I proteins in Huh-7 human cells (29).

Due to the importance of cholesterol for arboviral replication, pathogen blocking by *Wolbachia* in mosquito cells may be mediated by competition between *Wolbachia* and the arbovirus over cholesterol, its metabolites, and its precursors (30). In limited cholesterol conditions, *Wolbachia* has been shown to out-compete *Drosophila* C virus infection, impacting its replication in flies (30). This inhibition is not as effective when cholesterol is in abundance (30). Additionally, *Wolbachia*-mediated inhibition of virus activity could be due to bacteria-induced interference of intracellular cholesterol trafficking, disturbing replication complex formation or LD access (12).

Cholesterol biosynthesis is mediated by the mevalonate (MVA) pathway, which produces the terpenoid backbone precursors of sterols and isoprenoids in eukaryotic cells (31). In host cells, the MVA pathway is comprised of five enzymatic reactions, where the initial substrates of the pathway are acetyl-CoA and pyruvate, both of which are glycolytic products (32). The most important and well-studied enzyme in the pathway is 3-hydroxy-3-methyglutaryl-coenzyme A reductase (HMGCR), the rate limiting step that produces mevalonate. In parallel, *Wolbachia* uses glycolytic products for its own benefit (33). For example, *Wolbachia* from filarial nematodes (*Brugia malayi*) relies on host glycolysis and its glycolytic metabolites (e.g., pyruvate) (34). Glycolytic metabolites such as acetyl-COA and pyruvate can be redirected to bacteria, thus reducing their availability as substrates for cholesterol biosynthesis in *Wolbachia-*positive cells. Therefore, the division between the host MVA pathway and the needs of the bacteria forces sharing of these substrates between the host cell and the endosymbiotic bacteria. Consequently, there is potential for the bacteria to alter the activity of the MVA pathway to produce isoprenoids and cholesterol precursors.

In this study, we determined the impact *Wolbachia* has on regulating the balance between the MVA pathway and its own use of these resources. Additionally, we tested the effects on ZikV replication in *Aedes albopictus* C6/36 mosquito cells. We found that *Wolbachia* likely diverts resources, resulting in a decrease in abundance of cholesterol and LDs within the *Wolbachia*-infected mosquito cells. Functional analysis showed that alteration of cholesterol metabolism through either terpenoid backbone biosynthesis or cholesterol-esterase mechanisms inhibited ZikV infection in C6/36 cells, simulating *Wolbachia*-like effects.

## RESULTS

### Inhibition of the host MVA pathway increases *Wolbachia* loads and decreases Zika virus replication in mosquito cells

**Fig 1:**
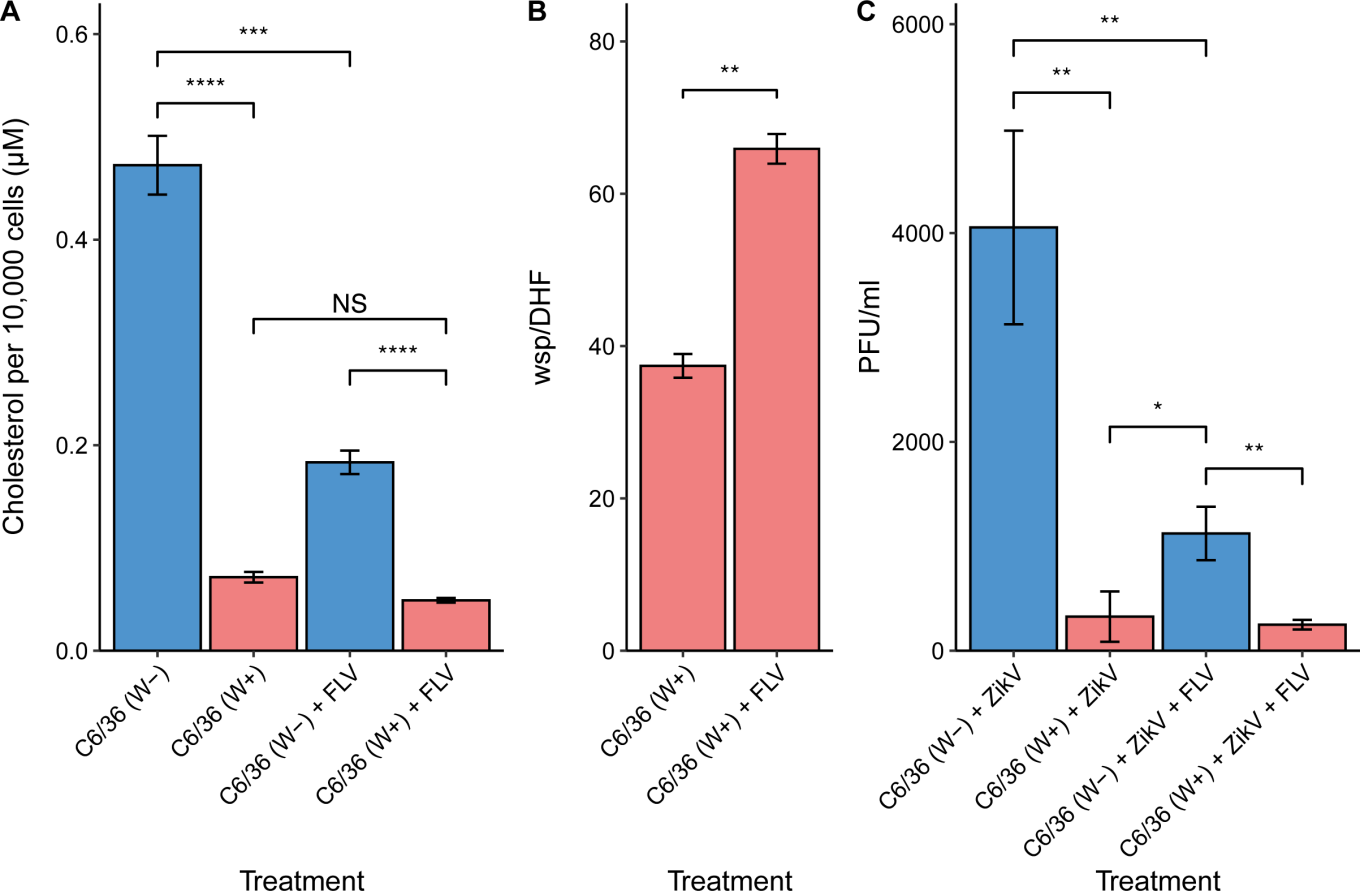
Effects of fluvastatin treatment on intracellular cholesterol levels, *Wolbachia* loads, and ZikV titers in C6/36 cells (4 days post-infection). (**A**) Intracellular cholesterol detected per 10,000 cells quantified by enzymatic cholesterol assay (Promega). (**B**) *Wolbachia* loads per C6/36 cell were measured via qPCR. (**C**) ZikV titers determined via plaque assay from supernatant collected from cultures at day 6 post-infection. Blue bars represent W− C6/36 cells while red bars represent W+ C6/36 cells.

To test the effects of *Wolbachia* and ZikV on host cholesterol biosynthesis through the MVA pathway, we studied cholesterol levels in *Wolbachia-*positive (W+) C6/36 cells compared to *Wolbachia-*free (W−) C6/36 cells to obtain a baseline for both conditions. We observed that cholesterol levels measured by enzymatic assay were significantly lower in W+ cells as compared to W− C6/36 cells (*P*-value <0.0001) (Fig. 1A). To investigate the discrepancy in cholesterol level in W− and W+ cells, we focused on the effects of *Wolbachia* and ZikV infection on cholesterol biosynthesis precursors produced in the MVA pathway. Fluvastatin (FLV) inhibits HMGCR, the rate limiting step of the host MVA pathway, and was used for *in vitro* treatment of C6/36 cells. In C6/36 W− cells where HMGCR was inhibited by FLV (3 µM), cholesterol level was significantly decreased compared to untreated cells (*P*-value <0.001) and remained at a low level in control and treated W+ cells (Fig. 1A). Besides the effects on cholesterol, FLV treatment in W+ cells increased the number of *Wolbachia* per cell (*P*-value <0.01) (Fig. 1B). We also evaluated the effects of HMGCR inhibition on ZikV replication in the C6/36 cells. In W− cells, ZikV titers significantly decreased after FLV treatment as compared to untreated cells (*P*-value <0.01). ZikV titers in W+ cells remained low and there was no significant difference between cells treated with FLV and untreated cells (Fig. 1C). These data indicate that inhibition of the host MVA pathway with FLV likely resulted in increased availability of resources for *Wolbachia*, leading to better *Wolbachia* replication. Conversely, inhibition of the host MVA pathway appeared to impact viral infection of C6/36 cells.

### Zika virus infection stimulates terpenoid backbone biosynthesis

**Fig 2:**
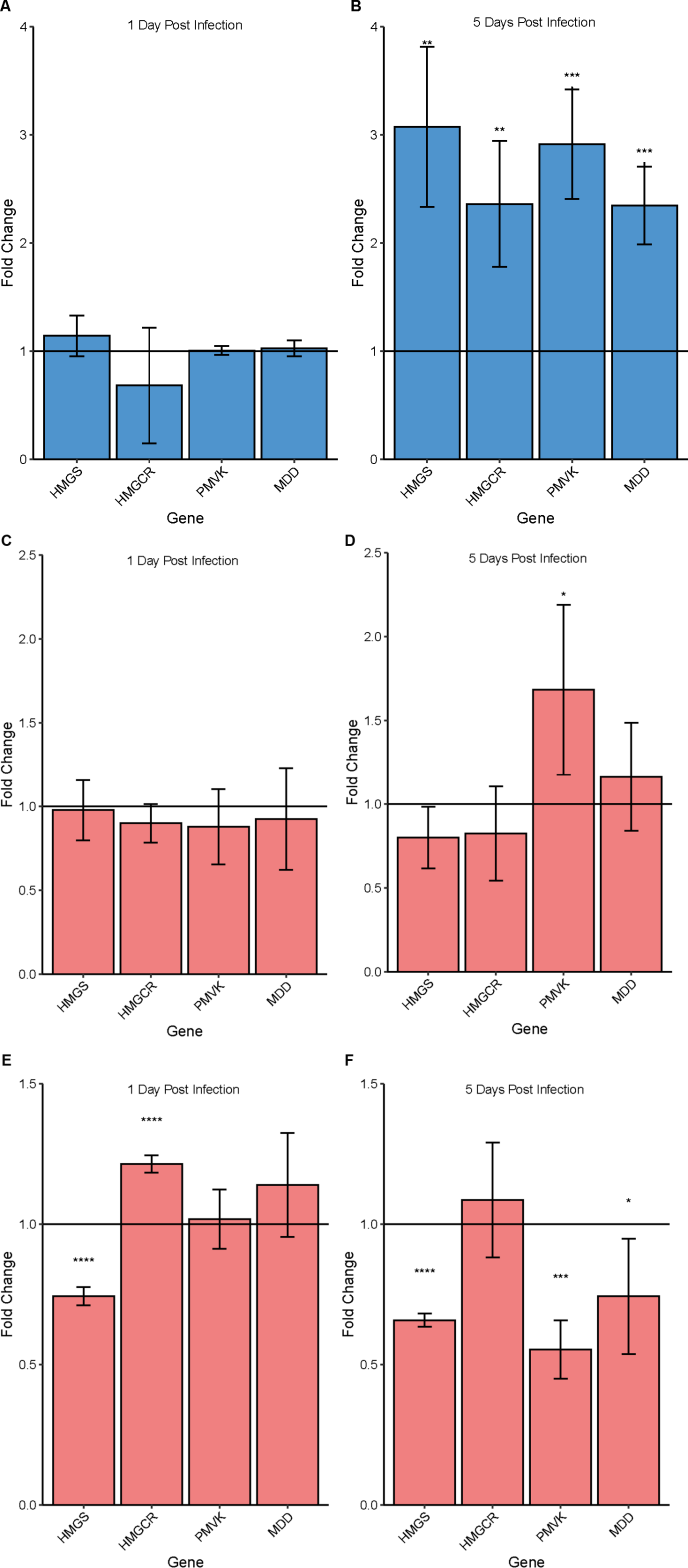
Fold change in MVA pathway gene expression in C6/36 cells. (A, B) Fold change in MVA pathway gene expression in response to ZikV infection in W− C6/36 cells at 1 dpi (**A**) and 5 dpi (**B**). Statistical significance was determined by comparing MVA pathway genes of interest to control samples (non-infected W− C6/36), which represents the horizontal line at y = 1. (C, D) Fold change in MVA pathway gene expression in response to ZikV infection in W+ C6/36 cells at 1 dpi (**C**) and 5 dpi (**D**). Statistical significance was determined by comparing MVA pathway genes of interest to control samples (non-infected W− C6/36), which represents the horizontal line at y = 1. (E, F) Fold change in MVA pathway gene expression in W+ C6/36 cells as compared to W− C6/36 cells. Horizontal line at y = 1 represents W− C6/36 data. Gene expression was determined via qRT-PCR and fold change values were calculated based on ∆∆Ct. HMGS, hydroxymethylglutaryl CoA synthase; HMGCR, 3-hydroxy-3-methylglutaryl-CoA reductase; PMVK, phosphomevalonate kinase, MDD, diphosphomevalonate decarboxylase. *P*-value <0.05 (*); *P*-value <0.01 (**), and *P*-value <0.001 (***).

Since we observed that inhibition of the MVA pathway led to decreased ZikV replication in C6/36 cells, we tested the effects of virus infection on the MVA pathway gene expression by quantitative real-time (qRT-PCR). We found that ZikV infection of C6/36 mosquito cells increased expression of all genes in the MVA pathway. At 1 day post-infection (dpi) with ZikV, host cell MVA pathway genes were not significantly differentially expressed as compared to uninfected controls for both W− (Fig. 2A) and W+ (Fig. 2C) cells. By 5 days post-infection with ZikV, the fold change in gene expression for all genes involved in the MVA pathway was significantly increased in W− cells (Fig. 2B) but not in W+ cells, except for phosphomevalonate kinase (AALF024095) (Fig. 2D). In contrast, the expression of MVA genes in W+ compared to W− cells did not show a consistent upregulation pattern at d1 or d5 in culture (Fig. 2E and F)

### *Wolbachia* and Zika virus infection decreases lipid droplets in C6/36 cells

**Fig 3:**
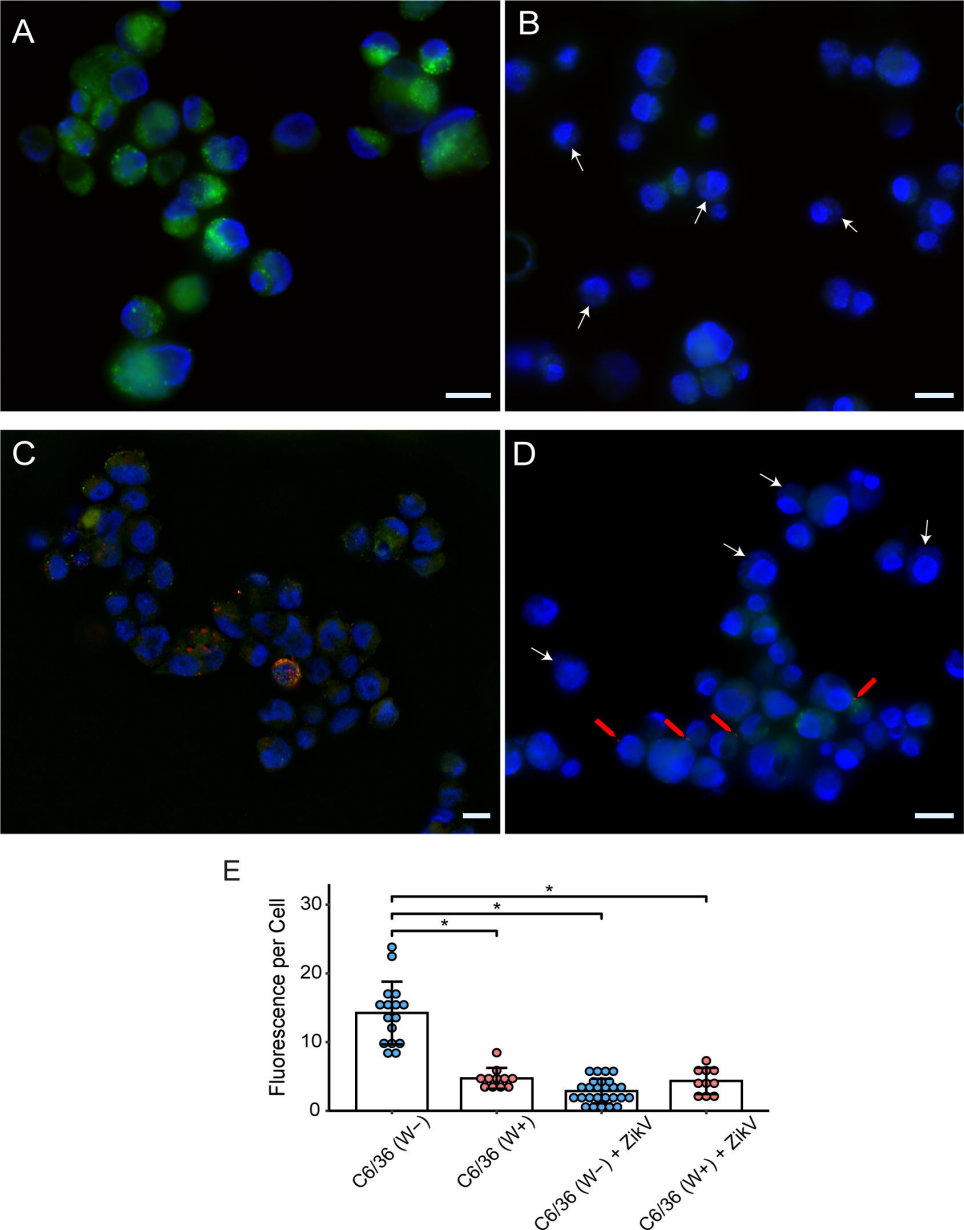
Fluorescence microscopy of lipid droplets and ZikV in W− and W+ C6/36 cells. Green fluorescence represents staining of lipid droplets with BODIPY; blue fluorescence highlights cellular DNA stained by DAPI (4′,6-diamidino-2-phenylindole); red fluorescence represents ZikV (anti-NS2B). (**A**) C6/36 (W−) cells present a strong green signal (BODIPY). (**B**) C6/36 (W+) cells; arrows indicate presence of small blue dots representing *Wolbachia* in cells. (**C**) C6/36 (W−) cells infected with ZikV (red). Green fluorescent signal is significantly lower. (**D**) C6/36 (W+) cells infected with ZikV (red, red arrow); arrows indicate some cells with *Wolbachia*. Scale bar is 10 µm. (**E**) Quantification of total fluorescent signal collected from W+ and W− cells with and without ZikV infection. Total fluorescent (green) signal per single cell was measured using ImageJ. Each point represents an average of fluorescent signal measurements from 10 to 20 individual cells. Blue points represent W− cells; red points identify W+ samples. *P*-value <0.05 (*).

After showing that terpenoid backbone biosynthesis through the MVA pathway is essential for virus replication, we next investigated an alternative source of cholesterol within host cells that could be used by ZikV. In eukaryotic cells, cholesterol is stored within LDs as cholesterol esters (CE), which can be converted to cholesterol by a cholesterol esterase. We first analyzed lipid droplet abundance in cells, followed by the expression of cholesterol esterase genes in C6/36 cells to determine how *Wolbachia* and ZikV alter host cholesterol metabolism. Using BODIPY staining, we examined lipid droplet characteristics in C6/36 cells infected with *Wolbachia* and ZikV. BODIPY is a common fluorescent dye for staining neutral lipids (including triacylglycerides and cholesterol esters) within lipid droplets (35). Lipid droplet size and abundance were significantly decreased in W+ C6/36 cells as compared to W− cells (*P*-value <0.05) (Fig. 3A, B, and E). Total fluorescent signal was measured by ImageJ, and there was a significant decrease of total fluorescent signal of lipid droplets post-ZikV infection for both W− and W+ cells as compared to uninfected C6/36 W− cells (*P*-value <0.05) (Fig. 3C through E). Besides LD depletion in ZikV+ cells, W+ cells experienced no other significant changes to cell morphology or health when comparing ZikV-infected and non-infected cells. While the reasons for lipid droplet depletion in *Wolbachia* and ZikV-infected cells may vary, these results show that *Wolbachia* infection significantly decreases availability of an alternative source of cholesterol contained in lipid droplets in mosquito cells.

**Fig 4:**
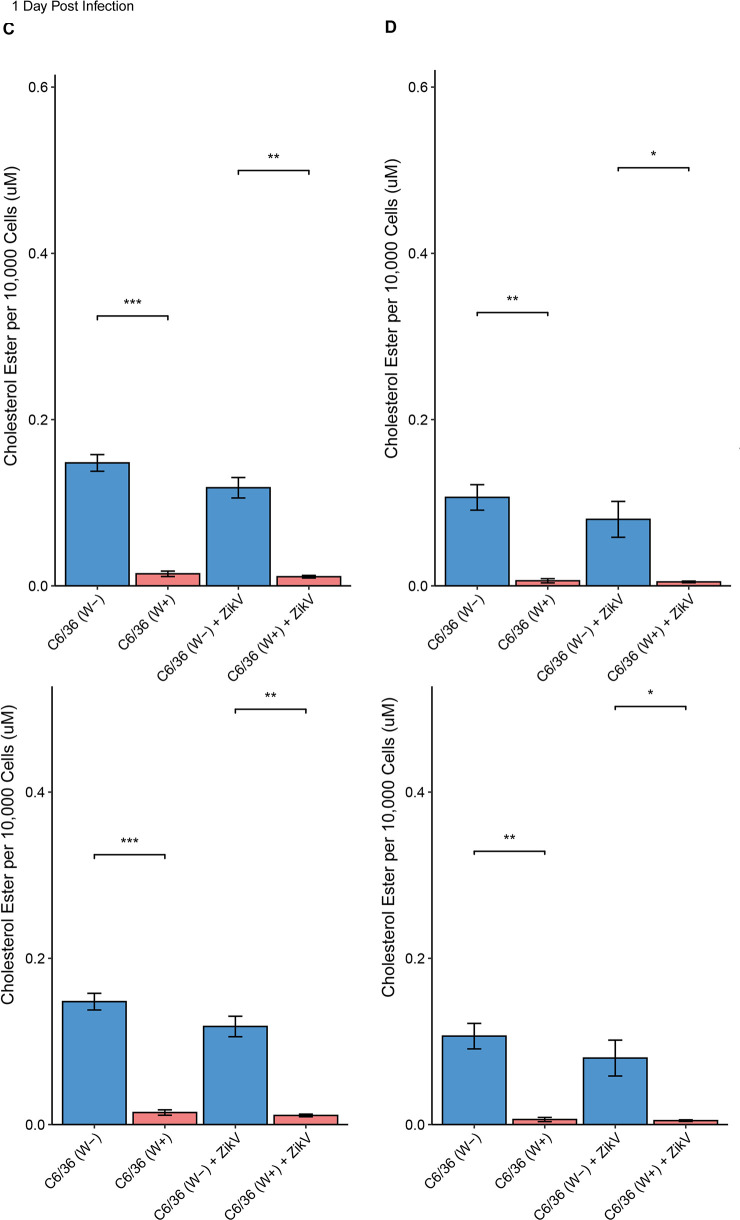
Effects of *Wolbachia* and ZikV infection on total intracellular cholesterol (**A and B**) and cholesterol ester (**C and D**) in C6/36 cells. Total cholesterol and cholesterol ester levels were determined via cholesterol assay on day 1 (**A and C**) and day 4 (**B and D**) post-ZikV infection. The concentrations were normalized by the average cholesterol per 10,000 cells. Blue bars represent W− samples while red bars represent W+ samples. *P*-value <0.05 (*), *P*-value <0.01 (**), and *P*-value <0.001 (***).

In addition to BODIPY staining, *Wolbachia-*mediated lipid droplet depletion was further corroborated via enzymatic cholesterol assay. We used cells collected on day 1 and day 4 post-infection to analyze the effects of infection on cholesterol concentration in W+ and W− cells. In W+ cells, abundance of total cholesterol was significantly lower as compared to W− cells (*P*-value <0.001) (Fig. 4A and B). There was no significant change in cholesterol level between days 1 and 4 post-infection for these samples. While we found that the abundance of neutral lipids was lower in ZikV-infected samples (Fig. 3E), the cholesterol assay showed an elevation in cholesterol levels between ZikV-infected and uninfected C6/36 W− cells at 1 dpi and 4 dpi; however, it was not statistically significant (Fig. 4A and B). Moreover, W+ cells contained low levels of total cholesterol, and there was no significant difference in total cholesterol levels between W+ cells infected with ZikV as compared to those not infected (Fig. 4). Cholesterol ester, the component of LDs, was also significantly lower in W+ cells as compared to W− cells. ZikV infection did not significantly change cholesterol ester concentration in C6/36 (W− or W+) cells (Fig. 4C and D).

### Zika virus infection impacts cholesterol esterase expression in C6/36 cells

**Fig 5:**
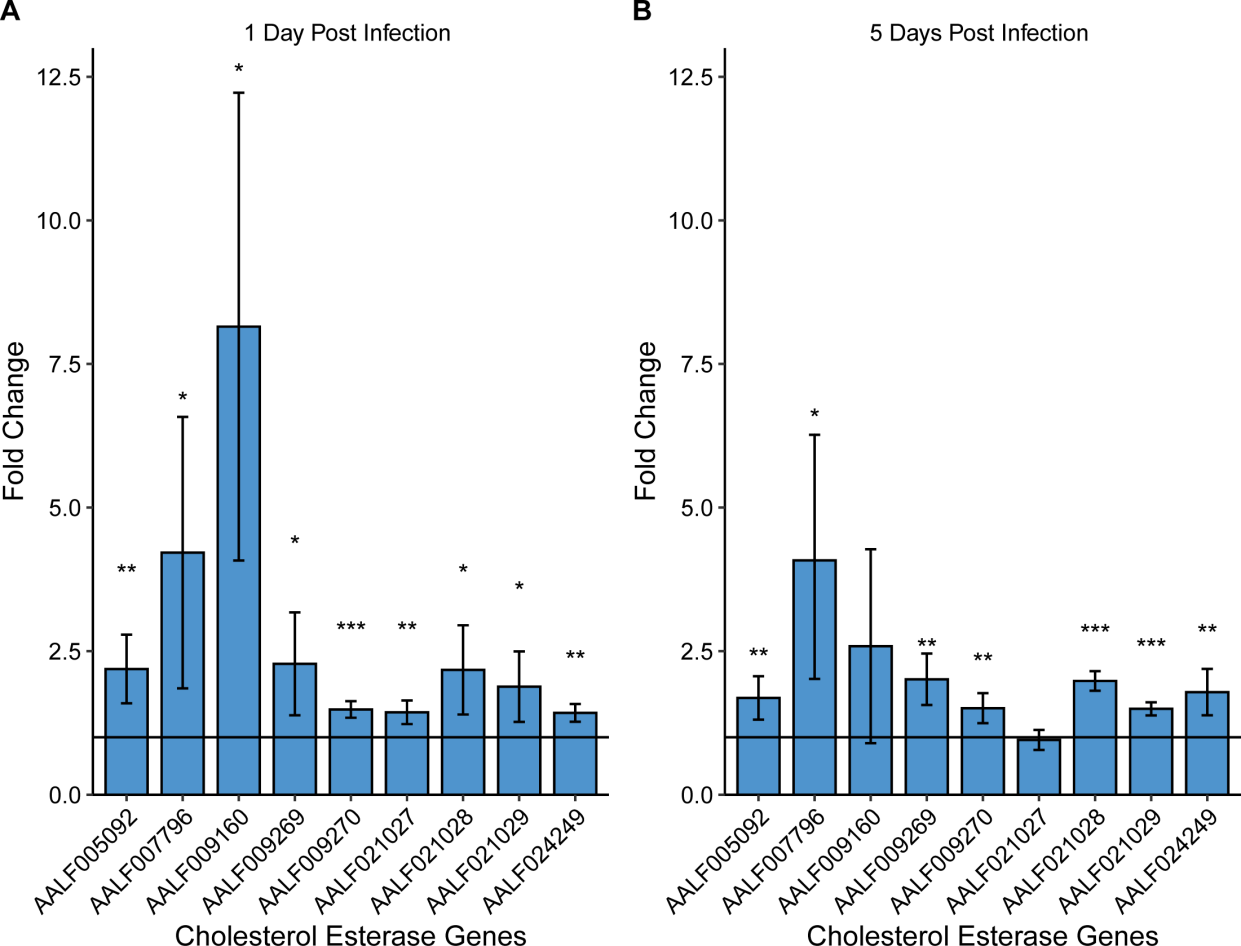
Fold change in cholesterol esterase gene expression after 1 dpi (**A**) and 5 dpi (**B**) with ZikV in W− C6/36 cells. All cholesterol esterase genes are homologs identified in *Ae. albopictus*. Gene expression was determined by qRT-PCR. Statistical significance was determined by comparing genes of interest to control samples (non-infected W− C6/36), which represents the horizontal line at *y* = 1. *P*-value <0.05 (*), *P*-value <0.01 (**), and *P*-value <0.001 (***).

To investigate the gene expression of cholesterol esterases during ZikV infection, we first searched for *Ae. albopictus* homologs of cholesterol esterase and then analyzed their expression by qRT-PCR in W− C6/36 cells infected with ZikV. We found nine genes predicted to function as cholesterol esterases in the genome of *Ae. albopictus* (Table S1). One day post-ZikV infection, all nine genes had increased expression as compared to the uninfected control (Fig. 5A), while after 5 days post-infection, seven of these nine genes continued to show significantly increased gene expression in ZikV-infected cells (Fig. 5B). The two genes that were not upregulated were lipases (AALF009160 and AALF021027). In W+ cells, there was no significant difference in cholesterol esterase gene expression 1 day post-ZikV infection as compared to the no virus control cells (Fig. S1A). However, all CE homologs were downregulated in W+ cells after 5 days post-ZikV infection as compared to no virus control cells (Fig. S1B). We also compared the expression of CE genes in W+ cells vs W− cells without viral infection. There were no genes significantly differentially expressed in W+ cells as compared to W− on day 1 of culture (Fig. S2A), but after 5 days in culture, eight of these nine genes showed significant increased gene expression (Fig. S2B).

### Dengue virus induces expression of cholesterol esterases in C6/36 cells, while *Wolbachia popcorn* prevents dengue virus-mediated changes

As observed for ZikV, DENV also hijacks host lipid metabolism during virus replication. Using publicly available transcriptomic data collected from C6/36 cells with a different strain of *Wolbachia* (*w*Melpop) and infected with DENV (36), we analyzed the gene expression of the nine homologs of cholesterol esterase in W− and W + cells (Table S1). After DENV infection of W− cells, eight of the nine genes had significantly higher expression compared to uninfected W− cells (*P*-value <0.05) (Table S2). The ninth gene (AALF009270) did have increased expression in DENV+ cells as compared to uninfected cells; however, the expression was not statistically significant. Interestingly, when comparing W+ cells with and without DENV infection, no cholesterol esterase genes were significantly differentially expressed between the two conditions (Table S2). This confirms that DENV and ZikV do not change the expression of cholesterol esterase in W+ C6/36 cells but do so in W− C6/36 cells (Table S2).

## DISCUSSION

In this study, we sought to determine *Wolbachia* mediated alterations to mosquito cholesterol metabolism that suppress ZikV replication. We show that *Wolbachia* infection significantly decreases total cholesterol level in mosquito cells. We predicted that bacteria subvert resources for cholesterol biosynthesis from the MVA (eukaryotic) pathway to fulfill bacterial needs. Suppression of the host MVA pathway by fluvastatin (FLV) significantly bolsters *Wolbachia* numbers in C6/36 cells, as compared to untreated controls. This indicates that the resources normally consumed in the MVA pathway were diverted to *Wolbachia,* driving bacterial replication. We also found that ZikV infection induced upregulation of genes involved in the MVA pathway of the mosquito cell. This indicates that the virus incites cholesterol biosynthetic processes that are necessary for viral replication (Fig. 2). This was validated by blocking the MVA pathway with FLV treatment, which led to a decrease in cholesterol levels and viral titers within the cell (Fig. 1A and C). In other flaviviruses, including DENV, MVA inhibition with statins has been shown to disrupt viral particle trafficking, leading to a decline in viral titers (37 – 41). Previous studies have proposed that cholesterol levels are involved with the ability of *Wolbachia*-infected *Drosophila melanogaster* to resist *Drosophila* C virus infections (30). In this study, we describe a mechanism of source depletion as *Wolbachia* competes for resources used in the MVA pathway, which indirectly impacts ZikV replication because of lower resources available via that same pathway.

In mosquito cells, the MVA pathway yields isopentenyl pyrophosphate (IPP), which is a secondary metabolite used for cholesterol synthesis in eukaryotic cells, yet it is not clear how *Wolbachia* uses these substrates. Studies of *Wolbachia* in *B. malayi* filarial nematodes showed that the bacteria use host glycolysis to obtain pyruvate for its survival (34, 42). Conditional experiments showed that added pyruvate increases bacterial numbers in worms and can be used in bacteria energy production and gluconeogenesis (34). Bacteria have the methylerythritol phosphate (MEP) pathway, which is an alternative to the eukaryotic cell MVA pathway, to produce IPP. Since *Wolbachia* infection in mosquito cells leads to cholesterol depletion, we can infer that MEP pathway activity in *Wolbachia* does not produce IPP or other secondary metabolites needed to sustain cholesterol levels detected in *Wolbachia-*free cells (Fig. 1A). Unfortunately, it is not possible to distinguish the distribution of glycolytic metabolites (such as pyruvate, acetyl-CoA) between eukaryotic cells and their symbiont to better evaluate the distribution of the resources within the cells. However, we believe that *Wolbachia* is a consistent consumer of carbohydrates and can redirect these resources.

**Fig 6:**
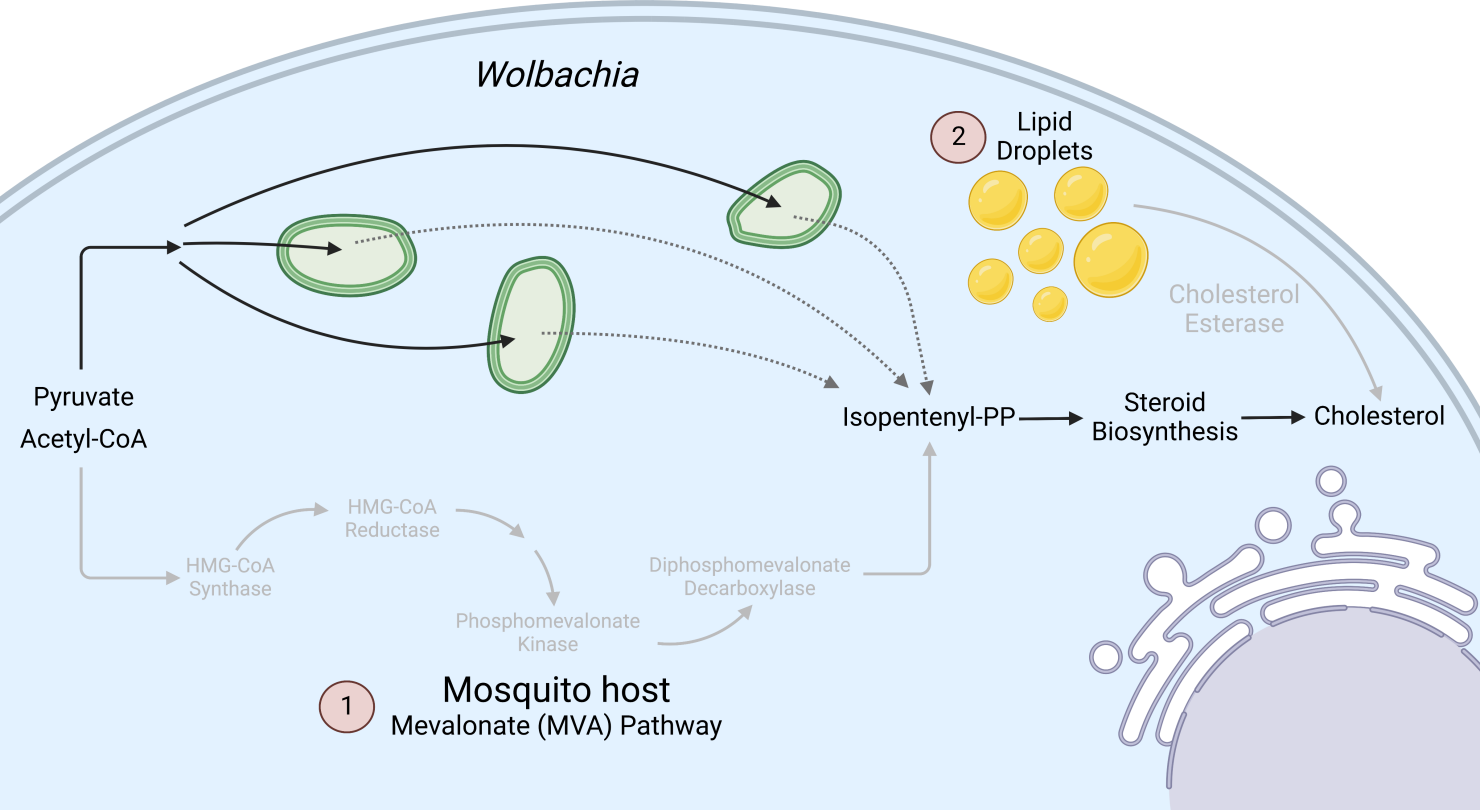
Schematic of the effects of how *Wolbachia* interferes with cholesterol metabolism to suppress Zika virus in C6/36 cells (1). *Wolbachia* uses the same substrates as the mosquito MVA pathway, leading to cholesterol depletion, and prevents ZikV upregulation of MVA genes. Light gray arrows indicate decreased expression in *Wolbachia-*infected cells (2). *Wolbachia* infection prevents ZikV from upregulating cholesterol esterase to use stored cholesterol in lipid droplets by decreasing LD abundance.

Decreased cholesterol in *Wolbachia*-infected cells indirectly suggests that bacteria need these resources (Fig. 6). This assumption is corroborated by findings in other obligate intracellular bacteria, including close relatives of *Wolbachia* (13). The tick-borne pathogens *Anaplasma phagocytophilum* and *Ehlichia chaffeensis* are intracellular bacteria that rely on stored host cholesterol and cholesterol uptake because of their lack of cholesterol biosynthesis capabilities (13, 43). These results show that *Wolbachia* disturbs cholesterol metabolism within the host cell, which decreases the efficiency of viral replication.

*Wolbachia-*mediated disruption of host cholesterol metabolic processes impacts lipid droplets, which are vital for viral particle assembly, formation, and trafficking to the ER, which has implications for replication complex formation (25). We determined how *Wolbachia* alters cholesterol storage by investigating cholesterol esterase activity. We found that cholesterol esterase activity was upregulated in ZikV-infected cells 1 day post-infection and continued over the course of the infection (Fig. 5). Our results show that over the course of ZikV infection, cholesterol esterase genes are upregulated, which is associated with LD depletion (Fig. 3). The upregulation of cholesterol esterase genes makes sense considering the *in vitro* conditions where cholesterol is limited in the media. As the virus continues to replicate, it creates a larger burden on the resources within the cell, causing the cell to upregulate these genes to release stored energy for its own cellular processes. We can infer that LD depletion in ZikV cells corresponds to a decrease in the cholesterol esters, triacylglycerides, and other lipids that comprise LDs. Interestingly, while cholesterol esterase genes are upregulated throughout the course of infection, cholesterol biosynthesis genes in the MVA pathway are not upregulated by the virus until later in the viral infection (Fig. 2). This is likely because by day 4 post-infection, viral particles have exited host cells, thus reducing the total amount of cholesterol and activating cholesterol esterase genes to maintain cholesterol levels within the desired range of the cell. In *Wolbachia* cells infected with ZikV, the virus was unable to upregulate these same genes (Fig. S1A). These results indicate that while hijacking by the virus of lipid droplets for cholesterol is important for virus replication and activity, the presence of *Wolbachia* prevents the virus from upregulating the cholesterol esterases needed to enhance viral replication. These alterations to cellular cholesterol metabolism by *Wolbachia* prevent ZikV replication. It is important to note that *Wolbachia* resides in C6/36 cells, causing the depletion of lipid droplets and overall total cholesterol levels. This means that in *Wolbachia*-positive cells, there is no substrate available for cholesterol esterases to work on and, therefore, the expression of cholesterol esterase genes is not upregulated. ZikV is reliant on lipid droplets for the formation of its capsid protein and uses resources from lipid droplets while synthesizing replication complexes (25). Since lipid droplet depletion in *Wolbachia-*infected cells decreases sources of cholesterol, ZikV is unable to use these resources to the same extent as in *Wolbachia-*free cells, therefore decreasing viral replication. We can conclude from these results that *Wolbachia* dampens viral-mediated upregulation of cholesterol esterase genes in mosquito cells, preventing viral access to important cholesterol stored in LDs. This study thus provides a deeper understanding of the pathogen blocking mechanism used by *Wolbachia*.

*Wolbachia-*induced changes to cholesterol esterase gene expression were also observed in DENV-infected C6/36 cells, with and without *w*Melpop. We found that DENV infection upregulated cholesterol esterase gene expression as compared to no virus controls in *Wolbachia*-free cells. On the other hand, there was no significant differential cholesterol esterase gene expression between *Wolbachia-*infected and *Wolbachia*-free cells. Cells infected with both DENV and *w*Melpop also did not significantly differ from *w*Melpop-infected cells. These results show similar findings to what we observed in our system with ZikV. This indicates that *Wolbachia* prevents both ZikV and DENV from upregulating cholesterol esterase genes that help the virus access cholesterol for enhanced replication.

Cholesterol depletion was found to interfere with intracellular replication and viral entry of other flaviviruses, including Japanese encephalitis virus and DENV (44). While cholesterol depletion plays a large role in *Wolbachia*-mediated viral inhibition, other studies have shown that cholesterol supplementation increases ZikV entry into *Wolbachia* (*w*Stri)-infected cells, although virus replication is not fully recovered (45). This implies that *Wolbachia* has a larger impact on viral suppression beyond depleting the cell of cholesterol. Other ways that *Wolbachia* may suppress viral replication is by disturbing lipid metabolism and trafficking, thus preventing virus replication complex formation (38) or viral entry into the cell (7, 45, 46). Others have found that acylcarnitines, a class of lipids, are altered in mosquitos by the presence of *Wolbachia* (47). Acylcarnitines have been found to help provide energy for the cell by transporting long-chain fatty acids into mitochondria (48), which leads to *B-*oxidation, found to be important for flavivirus replication (49). In *Aedes aegypti* mosquito cells, acylcarnitines have been found to be decreased in *Wolbachia-*containing cells, and depletion of acylcarnitines demonstrated increased *Wolbachia* density, while diminishing ZikV replication (47). This shows that the findings of our study represent only a portion of how *Wolbachia* modulates host cells to block ZikV growth within the cell, and that other metabolites also play an important role besides cholesterol and its derivatives.

Our study showed for the first time that *Wolbachia-*induced changes to both the mevalonate pathway and to cholesterol esterase activity are vital for ZikV infections (see model, Fig. 6). Understanding the mechanism of *Wolbachia*-induced interference with arboviruses will improve strategies to control arbovirus pathogens in vector populations. Moreover, insight gained in this study could also help better understand mechanisms to control virus dissemination in the human body, particularly as it relates to virus infection from mother to fetus and effects of viral infection on fetus development.

## MATERIALS AND METHODS

### Cells and virus

The *Aedes albopictus* mosquito cell lines C6/36 W+ and W− were provided by Dr. Benjamin Makepeace (University of Liverpool, UK) and were cultured in Schneider’s Drosophila medium (SDM, Gibco) containing 10% fetal bovine serum (FBS, HyClone) and 1% penicillin/streptomycin (Gibco). C6/36 cells were grown at 28°C in the presence of 5% CO_2_. *Wolbachia* infection in the cell line was confirmed and *Wolbachia* density was quantified using quantitative PCR (qPCR) by comparing the ratio of the *Wolbachia* surface protein gene (wsp) and the DHF (dihydrofolate reductase) *Ae. albopictus* gene (Table S3).

To measure ZikV titers, plaque assays were conducted using Vero (African green monkey kidney) cells that were obtained from the World Health Organization and were used between passages 141 and 149. Cells were cultured in Dulbecco’s modified Eagle’s medium (DMEM, Gibco) with 10% FBS (HyClone) and 1% penicillin/streptomycin (Gibco) at 37°C with 5% CO_2_. ZikV (Paraiba_01/2015) (50) stocks were grown in Vero cells and viral titer was determined via plaque assay.

### Cell infection and drug treatment

A day before the infection of C6/36 cells with ZikV, cells were split at a 1:4 ratio into 12.5 cm^2^ flasks (Falcon). Upon infection, old media were removed and pure SDM (Gibco) media with ZikV (MOI = 0.1) were added to the flasks for 2 h. During the 2-h infection, the flasks were gently rocked every 20 min. After the infection, complete media were added, and cells were maintained at 28°C with 5% CO_2_ until collection. Control cells were mock-infected with SDM media not containing ZikV.

During cell treatment with FLV, the compound was dissolved in DMSO (dimethylsulfoxide) and supplemented to SDM media with a final concentration of 3 µM. Control media were supplemented with DMSO with the same volume as the drug.

For each experiment, cells were collected day 1 post-infection (pi) and at either day 4 or 5 post-infection depending on the experiment. The cholesterol level was measured at the time when the virus initiates (d1) and expands (d4) formation of membranal intracellular components (ER, autophagosome, lysosome, other types of endosomes) where the virus replicates and forms. Virus formation requires membranes in the formation of the envelope. Therefore, cholesterol can be used at this stage. It peaks on day 4 pi in the cytoplasm of host cells. Gene expression was measured at day 5 to observe consistent changes in the host response to viral infection.

For plaque assay, the media of infected cells were collected on day 6 when most viruses have egressed from the cells. During collection, the supernatant of ZikV infection flasks was spun down to remove cellular debris and stored for future analysis. The cells were scraped and spun down for 3 min at 300 g. The collected cells were washed three times with PBS (phosphate-buffered saline, Gibco) before being aliquoted and stored at −80°C for future analysis.

### Cholesterol assay

To determine the effects of ZikV, *Wolbachia,* or FLV on C6/36 cell cholesterol levels, we used the luminescence-based Cholesterol/Cholesterol Ester-Glo Assay (Promega) following the manufacturer’s instructions. Total cholesterol was normalized by the number of cells estimated per sample aliquot.

### Plaque assay

ZikV titer was measured via plaque assay. Supernatant from ZikV infected cells was collected at the time of cell collection. Samples were diluted 1:10 in series to 10^−7^ of the original concentration per milliliter. After serially diluting this supernatant, 200 µL of each dilution was added to individual wells in a 24-well plate of 100% confluent Vero cells. The virus was left to infect the Vero cells at 37°C and 5% CO_2_ for 2 h, while gently rocking every 20 min. After the infection period, 2 mL of methyl cellulose overlay media [DMEM, 2% FBS, 1% penicillin/streptomycin (Gibco), and 1% methyl cellulose (Sigma)] were added to each well. Plaques were observed after 6 dpi, by staining vital cells with 1% crystal violet (Sigma) stain. Plaques were then quantified, and PFU per milliliter was calculated based on the number of dilutions.

### qRT-PCR

Quantitative real-time PCR was used to examine the expression of genes of interest related to terpenoid backbone biosynthesis and cholesterol esterase metabolism in all groups [C6/36 (W+ or W−) cells and infected or not with ZikV]. Total RNA of C6/36 cells was extracted using the RNAeasy Plus Mini-Kit (Qiagen), then cDNA was synthesized using the SuperScript IV (Invitrogen) kit using random hexamers. Primers for mosquito genes were designed using Primer Premier (4.1.0) (Table S3 and S4) and used with cDNA in qRT-PCR reactions conducted with a total volume of 20 µL using PowerTrack SYBR Green Master Mix (ThermoFisher Scientific) as the reporter. Expression was analyzed using the ddCt method using actin as the reference gene. Fold change expression was calculated based on control samples. All *Ae. albopictus* gene ID numbers were collected from VectorBase using the reference strain.

### Microscopy

Cells (4 dpi or control) were washed three times with PBS and then fixed with 4% formaldehyde in PBS. Cells were then washed with PBS and stained with anti-NS2B (ZIkV) protein (GeneTex) following incubation with secondary antibodies (ThermoFisher). After immunostaining, cells were stained with BODIPY 493/503 (ThermoFisher Scientific) for 1 h. After washing stained cells with PBS, they were mounted in Vectashield mounting media containing DAPI (for DNA staining) and analyzed under a Nikon Thunder microscope. For each sample, 10–30 images of different fields were taken to analyze the intensity of the BODIPY fluorescent signal per cell. Using ImageJ, images were converted to grayscale and total intensity per cell was calculated subtracting the background for each cell in the field. An average of the final intensity of the fluorescence signal was calculated per field and graphed while using *t*-test statistical analysis.

### Transcriptomic analysis

To assess the effects of arbovirus and *Wolbachia* infection on cholesterol esterase genes, we analyzed transcriptomic data from Teramoto et al. (36). The transcriptomic data from this study were from C6/36 cells in four treatments: control C6/36 cells, C6/36 cells infected with DenV, C6/36 cells infected with *w*Melpop, and C6/36 cells infected with both DenV and *w*Melpop. Each treatment had three biological replicates with the exception of the *w*Melpop-infected cells which had two biological replicates. We used VectorBase to identify cholesterol esterase homologs in *Ae. albopictus*. The DESeq2 R4.2.0 package was used to find the differential expression for each gene, where the log fold change and adjusted *P*-value were calculated.

### Statistical analyses

Statistical analyses were performed with PRISM 9.3.1 software (Graph Pad Inc). All experiments had at least three biological replicates and the comparisons between groups were made using non-parametric *t*-tests. A *P*-value <0.05 was considered to be statistically significant.

## SUPPLEMENTAL MATERIAL

Fig. S1 spectrum.02180-23-s0001.docx Fold change in cholesterol esterase gene expression after day 1 (A) and day 5 (B) in ZikV-infected W+ C6/36 cells as compared to uninfected W+ C6/36 cells.

Fig. S2 spectrum.02180-23-s0002.docx Fold change in cholesterol esterase gene expression after day 1 (A) and day 5 (B) in W+ C6/36 cells as compared to W- C6/36 cells.

Table S1 spectrum.02180-23-s0003.xlsx Homology of *Ae. albopictus* genes of interest to other model systems.

Table S2 spectrum.02180-23-s0004.xlsx Transcriptomic analysis of cholesterol esterase gene expression in C6/36 cells infected with dengue virus and wMelPop.

Table S3 spectrum.02180-23-s0005.docx Primers used in qRT-PCR for MVA pathway genes and for the determination of *Wolbachia* load per mosquito cell.

Table S4 spectrum.02180-23-s0006.docx Primers used in qRT-PCR for cholesterol esterase genes.

ASM does not own the copyrights to Supplemental Material that may be linked to, or accessed through, an article. The authors have granted ASM a non-exclusive, world-wide license to publish the Supplemental Material files. Please contact the corresponding author directly for reuse.
