# Supplementary material for: Wolbachia interferes with Zika virus replication by hijacking cholesterol metabolism in mosquito cells
Source: Microbiol Spectr. 2023 Oct 9;11(6):e02180-23. doi: 10.1128/spectrum.02180-23 (PMC10715073; doi:10.1128/spectrum.02180-23)
Supplement: Fig. S1 — Fold change in cholesterol esterase gene expression after day 1 (A) and day 5 (B) in ZikV-infected W+ C6/36 cells as compared to uninfected W+ C6/36 cells. [file spectrum.02180-23-s0001.docx]

**Supplemental File**


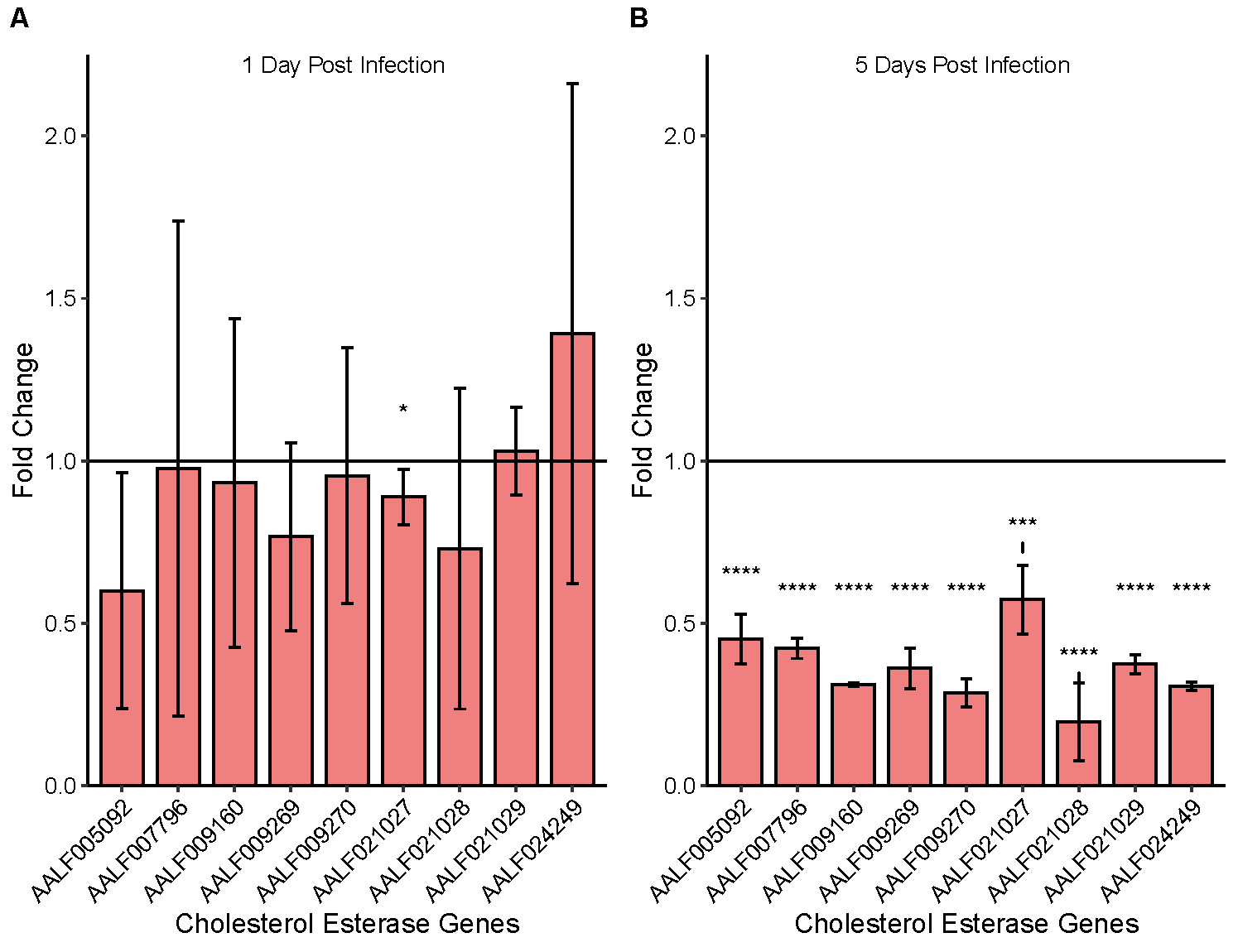


**FIG S1. Fold change in cholesterol esterase gene expression after day 1 (A) and day 5 (B) in ZikV infected W+ C6/36 cells as compared to uninfected W+ C6/36 cells.** All cholesterol esterase genes are homologs identified in *Ae. albopictus.* Gene expression was determined via qRT-PCR and fold change values were calculated based on ∆∆Ct. Statistical significance was determined by comparing genes of interest to uninfected W+ C6/36 cells, which is represented by the horizontal line at y = 1. P-value < 0.05 (*), p-value < 0.01 (**), and p-value < 0.001 (***).
