# Supplementary material for: Wolbachia interferes with Zika virus replication by hijacking cholesterol metabolism in mosquito cells
Source: Microbiol Spectr. 2023 Oct 9;11(6):e02180-23. doi: 10.1128/spectrum.02180-23 (PMC10715073; doi:10.1128/spectrum.02180-23)
Supplement: Table S3 — Primers used in qRT-PCR for MVA pathway genes and for the determination of Wolbachia load per mosquito cell. [file spectrum.02180-23-s0005.docx]

### **Supplemental File**

**Table S3.** Table of primers used in qRT-PCR for MVA pathway genes and for the determination of *Wolbachia* load per mosquito cell.

| **Gene: VectorBase ID** |  | **Sequence 5’ – 3’** |
| --- | --- | --- |
| ***Hydroxymethylglutaryl-CoA Synthase*: AALF023323** | Upstream  Downstream | TGTTGGAATTTTGGGGCTGG  GAGTTGACATCTTCGCGGTC |
| **Predicted HMGCR: AALF008540** | Upstream  Downstream | TACATCCTGATCATCGCGCT  GTCTCGGCTAGTGTGAAGGT |
| **Predicted PMVK: AALF024095** | Upstream  Downstream | AGTGGAGCGATCAGAAACGA  GTCCCCTCACTGGCTACAAT |
| ***Diphosphomevalonate Decarboxylase*: AALF005923** | Upstream  Downstream | TGGTGCATCAGTTCAACGTC  GTTCAGCCAAATCCACAGCA |
| **Actin (reference)** | Upstream  Downstream | GCAAACGTGGTATCCTGAC  GTCAGGAGAACTGGGTGCT |
| ***Wolbachia* WD0550** | Upstream  Downstream  ­­ | CAGGAGTTGCTGTGGGTATATTAGC  TGCAGGTAATGCAGTAGCGTAAA |
| ***A. albopictus DHF*** | Upstream  Downstream | GGAGTACCTGAGTCGAAGCG  GCACACCATCACCTCCGAT |
