# Supplementary material for: Wolbachia interferes with Zika virus replication by hijacking cholesterol metabolism in mosquito cells
Source: Microbiol Spectr. 2023 Oct 9;11(6):e02180-23. doi: 10.1128/spectrum.02180-23 (PMC10715073; doi:10.1128/spectrum.02180-23)
Supplement: Table S4 — Primers used in qRT-PCR for cholesterol esterase genes. [file spectrum.02180-23-s0006.docx]

**Supplemental File**

**Table S4.** Table of primers used in qRT-PCR for cholesterol esterase genes.

| **Gene: VectorBase ID** |  | **Sequence 5’ – 3’** |
| --- | --- | --- |
| ***Lipase*: AALF005092** | Upstream  Downstream | CCGGTGTTGCTGATACATGG  CATTTCCCAGCCAAACGTCA |
| ***Carboxylic Ester Hydrolase*: AALF007796** | Upstream  Downstream | CGTATCTGCATTTTCCGCCA  GACAGCTGCATCACCACTTC |
| ***Lipase*: AALF009160** | Upstream  Downstream | GCCCGTAGTTCTGCTGCAG  CCACGAGAAGTCCCAGAACT |
| ***Carboxylic Ester Hydrolase*: AALF009269** | Upstream  Downstream | TGGGATTCTTCAGCACTGGT  GATCACCACCAAAAGCTGCA |
| ***Carboxylic Ester Hydrolase*: AALF009270** | Upstream  Downstream | CAATACGGTTCCTCATCGCG  CCTCCCGAGAAAGATCCTCC |
| ***Lipase*: AALF021027** | Upstream  Downstream | CTAGCATACCTTCTCGCCGA  CATGGCGGGTAGATCGTAGT |
| ***Lipase*: AALF021028** | Upstream  Downstream | CCTTGTCCCCACCTGCA  CGGCCAACATGTAGACCAAG |
| ***Lipase 1 Precursor*: AALF021029** | Upstream  Downstream | ACAGCCTCGCCTACTTACTC  CTTGGCCGGTAGATCGTAGT |
| ***Lipase*: AALF024249** | Upstream  Downstream | GGGTTCGATGTTTGGCTACC  CCGGTAAGCCTCAGAACGTA |
| **Actin (reference)** | Upstream  Downstream | GCAAACGTGGTATCCTGAC  GTCAGGAGAACTGGGTGCT |
